# A Bird’s-Eye View of Endangered Species Conservation: Avian Genomics and Stem Cell Approaches for Green Peafowl (*Pavo muticus*)

**DOI:** 10.3390/genes14112040

**Published:** 2023-11-04

**Authors:** Sittipon Intarapat, Woranop Sukparangsi, Oleg Gusev, Guojun Sheng

**Affiliations:** 1 Department of Anatomy, Faculty of Science, Mahidol University, Bangkok 10400, Thailand; 2 Department of Biology, Faculty of Science, Burapha University, Chonburi 20131, Thailand; woranop@go.buu.ac.th; 3 Regulatory Genomics Research Center, Institute of Fundamental Medicine and Biology, Kazan Federal University, 420008 Kazan, Russia; o.gusev.fo@juntendo.ac.jp; 4 Intractable Disease Research Center, Graduate School of Medicine, Juntendo University, Tokyo 113-8421, Japan; 5 Life Improvement by Future Technologies (LIFT) Center, 143025 Moscow, Russia; 6 International Research Center for Medical Sciences, Kumamoto University, Kumamoto 860-0811, Japan; sheng@kumamoto-u.ac.jp

**Keywords:** avian conservation, peafowl, green peafowl, avian genomics, avian stem cells

## Abstract

Aves ranks among the top two classes for the highest number of endangered and extinct species in the kingdom Animalia. Notably, the IUCN Red List classified the green peafowl as endangered. This highlights promising strategies using genetics and reproductive technologies for avian wildlife conservation. These platforms provide the capacity to predict population trends and enable the practical breeding of such species. The conservation of endangered avian species is facilitated through the application of genomic data storage and analysis. Storing the sequence is a form of biobanking. An analysis of sequence can identify genetically distinct individuals for breeding. Here, we reviewed avian genomics and stem cell approaches which not only offer hope for saving endangered species, such as the green peafowl but also for other birds threatened with extinction.

## 1. Introduction

Approximately 12% of bird populations are threatened with potential extinction, according to the International Union for Conservation of Nature (IUCN) [1]. Efforts to conserve endangered wild birds can be put forth using different approaches, such as assisted reproductive technology (ART), biotechnological tools, and public awareness [2,3]. Alongside mammalian counterparts, several ARTs commonly performed in mammalian species were reported to be partially achieved in avian species, such as artificial insemination (AI), in vitro fertilization (IVF), embryo transfer (ET), intracytoplasmic sperm injection (ICSI), gonadal tissue transplantation, and the manipulation of avian embryonic cells [4,5,6,7,8,9]. However, the evidence for ICSI and IVF is weak and literally no healthy offspring have been hatched using these techniques. 

The advancement of the genomic era also allows researchers to assess genetic make-up, compare genetic diversity parameters between wild populations and captive species, and develop molecular markers as parameters for preserving genetic diversity and inbreeding issues [10,11,12,13]. The accessibility of these data can be advantageous for calculating harvest rates as well as managing or the translocation of wild birds for applications of wildlife management and conservation [5,14]. Importantly, genomic studies play a crucial role in conserving endangered species and studying endangered species populations that help to obtain more information regarding the effects of inbreeding, including the increase in genetic drift that leads to decreased genetic diversity in isolated populations in wild birds, particularly in green peafowl [14,15,16]. 

Stem cell-mediated technology using induced pluripotent stem cells (iPSCs) shows promise in avian reproductive biotechnology for rescuing endangered birds [17,18,19,20]. By deriving iPSCs from different types of somatic cells of endangered avian species, it becomes possible to preserve these cells in biobanks, enabling the generation of viable gametes for reproductive-assisted technology and conservation purposes [17,20,21,22,23,24]. This review discusses peafowl biology, highlighting the green peafowl as an endangered species model. Furthermore, we review avian genomics and stem cell approaches for conserving other endangered bird species. 

## 2. Peafowl and Green Peafowl

Peafowl, the largest and most vibrant birds in the genus Galliformes [25], are classified in Genus *Pavo* and *Afropavo* within the Family *Phasianidae* and Subfamily *Pavoninae*. The three existing species are Indian or blue peafowl (*Pavo cristatus*), green peafowl (*Pavo muticus*), and Congo peafowl (*Afropavo congensis*) [26]. These birds fascinate with their well-developed plumage, displaying a range of iridescent shades [27]. *P. cristatus*, the Indian peafowl, grows to an average size of 198–229 cm and acquires its magnificent train after three years. The tail’s long ornamental feathers are elongated covert feathers, not genuine rectrices. Found in India and Sri Lanka, they primarily inhabit dry deciduous lowland forests up to 1500 m above sea level. Its clutch size typically varies from 3 to 8 eggs, with an incubation period of 28–30 days [28]. *P. muticus*, known as the green peafowl, has a larger size (213–244 cm) compared to *P. cristatus*. Its defining features include a greenish neck, an impressively long train, striking green plumage, and blue-yellow facial skin. This species is native to various regions, including China, Assam, Burma, Thailand, Vietnam, Laos, Malaysia, and Java. It prefers open forests, riverbanks, and forest edges, extending its range to 1000 m. Similar to *P. cristatus*, the green peafowl has a clutch size and incubation period of the same duration [28]. *Afropavo congensis* or the Congo (African) peafowl is the smallest species measuring around 60–68 cm. It features distinctive traits like a dark glossy green and bronze back rump, dark-green underside, and iridescent violet back of the neck and sternum. Its crown exhibits white and black spots with blue facial skin. This species is native to the Congo basin’s dense lowland tropical rainforests. It lays 3–4 eggs in a clutch, and the incubation period is 26–27 days, shorter than *P. cristatus* and *P. muticus* [28].

**Figure 1:**
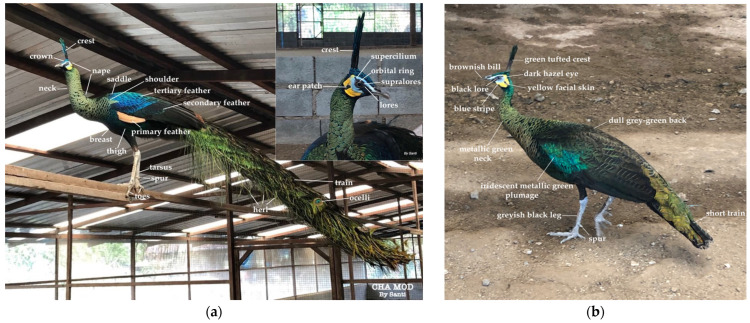

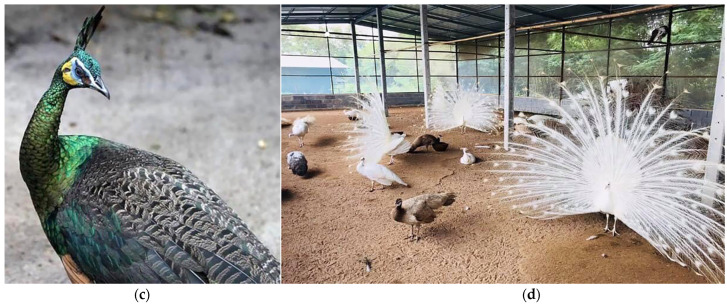
Phenotypic differences between green peacock (**a**), and green peahen (**b**) in comparison with hybrid peafowl (*P. muticus* × *P. cristatus*) (**c**) and plumage color variations (**d**). A mature male peacock displays a striking appearance after reaching the third year of age. They possess a small head with a turquoise blue color, slightly green, adorned with a compact crown of long blue–green feathers with a green tufted crest. The face is white with a blue stripe stretching from the base of the beak to the eye, while the area of the ear patch and lower jaw features a yellow crescent stripe are shown in the inset. The neck exhibits long and iridescent metallic-green feathers, and the breast and saddle are green with a metallic sheen. The primary feathers are light brown, while the secondary feathers appear bluish-grey. The shoulders and base of the wing have a grey–brown color with a light-blue–green sheen. The tarsus of the leg is in a greyish-black shade with spur found in both sexes. The primary color of the tertiary feather is yellow–green. Note that female peahen resembles a male with reduced iridescent plumage, a smaller neck, breast, and back scaly features, and a short train without ocelli and herl. (Source: courtesy of Santisak Thanomsing).

It is worth noting that the green peafowl, one of the world’s most beautiful birds (Figure 1), is a protected species in Thailand and holds significant value among the 39 pheasant species worldwide. Southeast Asia’s peacocks are divided into three subspecies based on plumage and distribution: Javanese green peafowl (*P. muticus muticus*), Indochina Peacock (Indo-Chinese green peafowl, *P. muticus imperator*), and Burmese green peafowl, *P. muticus specifer*. In Thailand, two subspecies can be found: Javan peacock or Southern peacock (*P. muticus muticus* Linnaeus, 1766) and Indochina peafowl or Northern peacock (*P. muticus imperator* Delacour, 1949) [29]. The green peafowl, listed as endangered on the IUCN Red List (http://www.iucnredlist.org) (accessed on 19 July 2023) and CITES Appendix-II, is experiencing a population decline [30]. In contrast, Congo and Indian peafowls are classified as vulnerable and least concern, respectively [26]. Habitat loss [31], inbreeding [15], and the poaching of feathers and flesh [30] contribute to the decline. 

Globally, climate change presents a challenge that impacts avian species [32]. As temperatures increase, concerns about declining bird populations have been raised in affected regions. Recent reports emphasize that 7% of bird species are now at risk of extinction due to the impacts of climate change [32]. In addition, avian species are regarded as important bioindicators of climate change [33]. Interestingly, it has been observed that the Galliformes order is particularly susceptible to an elevated risk of extinction because of increasing climate changes [34,35,36]. Climate change significantly contributes to shifts in the distribution of Pheasant species, primarily through the processes of habitat fragmentation and reduced connectivity [37,38]. Furthermore, the influence of climate change extends to the Peafowl species, marking it as one of the species affected by environmental changes [25]. 

What is the impact of climate change on Peafowl species? Climate fluctuations are anticipated to result in various outcomes, including shifts in the timing of peafowl breeding and migration, changes in the distribution of different peafowl species, variations in peafowl population sizes and species interactions, and significant alterations in community dynamics that lead to widespread shifts in Peafowl communities on a global scale [39]. It has been reported that the Indian peafowl may serve as an indicator of changing climate conditions [33]. Furthermore, changes in climate patterns have been linked to the diminishing green peafowl population [10,16]. Historical climate change has caused a significant reduction in their population as it has been indicated that climate-induced changes in range during the Pleistocene–Holocene transition had an impact on the green peafowl [16]. Notably, wild species have evolved as a response to shifts in climate conditions [40]. The complexity of understanding how various peafowl species can effectively adapt to different environmental scenarios in the context of climate change is crucial. This adaptation is particularly remarkable for the green peafowl, which currently faces significant declines in population and a reduction in its distribution range [41]. 

Taken together, anthropogenic influences and the crisis of climate change play pivotal roles in the green peafowl population decline that can lead to species extinction. However, understanding these factors remains an ongoing challenge that needs further investigation. To shed light on this complex relationship, emerging methodologies integrated with anthropogenic, climatic, and genomic data would offer a promising conservation plan. This multidisciplinary approach holds great potential to unravel multifactorial issues influencing biodiversity and future preservation strategies.

Preserving genetically purebred of green peafowl can involve a multi-faceted approach, encompassing the habitat conservation of endangered wild populations, education on the significance of species biodiversity, scientific investigation into interspecific hybridization, and recreating public awareness through visits to zoological parks [42].

## 3. Avian and Peafowl Development

Birds with more than 10,000 extant species are a closely related group of sauropsids (reptiles). Birds diverged from crocodiles, their closest reptilian relatives, about 240 million years ago [43]. However, neognaths (e.g., chickens and zebra finches) and palaeognaths (e.g., ostriches and emus), two major extant avian clades, diverged only about 110 million years ago [44], suggesting a narrow window of phylogenetic divergence among all living birds. This close relationship is reflected in avian development from fertilization to hatching. The main avian developmental features are well conserved (e.g., even between chickens and emus [45]), and standard staging systems describing chick development (Eyal-Giladi and Kochav (EGK) stages for pre-egg laying development [46]; and Hamburger and Hamilton (HH) stages for post-egg laying development [47]) can be applied to all living birds. The optimal temperature for egg incubation temperature varies slightly, and the duration of incubation varies significantly, even among closely related avian groups. Developmental speed and heterochrony become prominent at the mid-to-late phase of avian development. 

Both blue and green peafowl (*P. cristatus* and *P. muticus*, respectively) belong to the avian order, Galliformes, which also includes other well-known landfowl (e.g., chickens, quail, and turkeys). The peafowl lineage diverged from the junglefowls (including domesticated chickens) about 33 million years ago [48]. Both *P. cristatus* and *P. muticus* hatch after about 28 days of incubation (in comparison, chicken: 21 days; quail: 18 days; and turkey: 28 days). Their eggs resemble chicken eggs in coloration (light brown) and large duck eggs in size, with green *P. muticus* eggs being slightly larger than *P. cristatus* ones. Peafowl breeding season in Japan lasts from April to the end of July. Freshly laid blue peafowl eggs collected from Kumamoto City Zoo and Botanical Gardens, Kumamoto have a very high fertilization rate, whereas green peafowl eggs collected from Santi Peacock Farm have a low fertility rate, possibly reflecting their small founder population size and poor adaptation to a captive environment (personal communication). Peafowl embryos at oviposition are similar in developmental stage (EGK X–XI) and morphology to those of chicken. Embryonic fibroblast cells collected from blue peafowl also resemble chicken ones in their morphology and growth rate. Regular incubators for chicken eggs can be used for developmental studies and hatching. 

## 4. Avian and Peafowl Genomics and Genetics 

### Avian and Peafowl Omics Resources 

During the last decades, the rapid evolution of sequencing platforms has greatly contributed to the understanding of avian genome structures and boosted the usage of gene manipulation technologies for non-model organisms. Comparative genomics made significant progress based on the multiple genome comparison and deducting species-specific traits empowered by the multi-omics data [49]. Birds are among the most diverse groups of vertebrates combined with more than ten thousand species with complex and diverse morphology and physiology [50,51]. The genomes of birds are relatively compact which makes them attractive models for both evolutionary studies and in vivo/in vitro genomic manipulations. The first bird genome (domestic chicken *Gallus gallus*) was assembled in 2004, and currently, more than 800 genomes of birds have been sequenced and assembled with high quality (according to www.ncbi.nlm.nih.gov, accessed on 29 September 2023). Specifically, The Bird 10,000 Genomes (B10K) project ambitiously aims to decode the genomes of all existing bird species, currently climbing up to 2000 species sequenced to date. These efforts underline the importance of the birds’ genomics resources, and a number of the specialized engines focused on cross-genome comparison are growing rapidly (https://www.biomedcentral.com/collections/avian) (accessed on 29 September 2023).

The genome of birds is generally small, staying in the range from 0.91 Gb (black-chinned hummingbird, *Archilochus alexandri*) to 1.3 Gb (common ostrich, *Struthio camelus*) [52]. In addition to a generally smaller size, the bird genome is characterized by a lower number of repetitive elements [53,54], a low proportion of non-coding regions in general, and intensive evidence of gene loss [54]. Sex in birds is specified genetically, with the female being heterogametic (ZW sex chromosomes) and male homogametic (ZZ sex chromosomes) in contrast to eutherian mammals [55,56]. Phylogenomic analyses indicated that chrZ and chrX evolved separately from different autosomes, and likely originated from the dimorphic expression of autosomal genes involved in sex differentiation [57]. We have recently showed that three-fourths of chrZ genes are strictly compensated across Aves, similar to mammalian chrX chromosomes [58].

In the light of the complexity of avian genomes and biology, as well as incomplete annotation for many species, it is still challenging to effectively modify birds’ genomes using genome modification tools [59,60,61]. Traditionally, the approaches are built around the genome editing of primordial germ cells (PGCs) and transplantation of the PGCs into recipient embryos [62,63]. Recently germline mosaic founder chicken and duck lines without the PGC-mediated procedures by injecting an adenovirus containing the CRISPR-Cas9 system into avian blastoderms were obtained, demonstrating successful applications of the adenovirus-mediated method for the production of genome-edited chicken and duck lines, opening new horizons for active use of genome modification technologies in poultry [64]. The effective use of genetic technologies in birds would require further advances in genome annotations, including the systematic characterization of non-coding RNA and transcribed regulatory elements (promoters and enhancers) for birds’ genomes, as is performed for mammals, including humans, in the frame of ENCODE, FANTOM5 and GTEx consortiums [65]. We recently launched an ambiguous project, Functional Annotation of Animal Genomes (FAANG), aiming for the systematic characterization of regulatory elements in birds’ genomes, and we expect steady progress in this field [66]. In addition to chicken, we have recently demonstrated the power of cross-species transcriptomics resources in identifying reliable biomarkers and tracing the evolution of regulatory elements in birds [58].

There is still the lack of a well-annotated genome of green peafowl, but ongoing efforts give hope to fill this gap soon [12]. A draft genome of Indian peafowl with a size of 1.05 Gbp was assembled and partially annotated [13]. It was concluded that Indian peafowl shows remarkable changes compared to other birds in terms of genes associated with metabolism, immunity, and skeletal and feather development [13]. Surprisingly, a comparison of genomic data from green and blue peafowl suggests drastic differences in genome structure, including gene number in these two species [67], making this group of birds an interesting model for evolutionary changes in closely related species.

## 5. Avian and Peafowl Stem Cell Biology

### Approaches for Applying Stem Cell Research in Peafowl Conservation 

**Figure 2:**
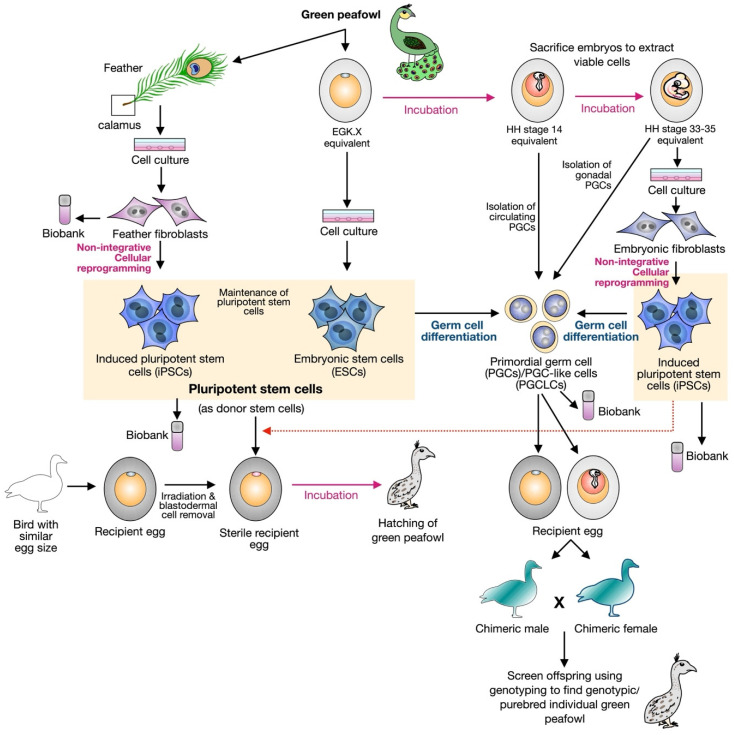
Strategy for preserving green peafowl using stem cell technology. Tissue from the calamus area of a green peafowl feather can be collected for primary culture to obtain feather fibroblasts, which can then be used for cellular reprogramming without genome modification to generate induced pluripotent stem cells (iPSCs). Alternatively, blastodermal cells from a stage equivalent to the chicken embryo EGK X stage can serve as a source to obtain embryonic stem cells (ESCs). Fertile eggs can also be further incubated to reach a stage equivalent to the chicken HH stage 14 for the isolation of circulating primordial germ cells (cPGCs), or a stage equivalent to chicken HH stage 33–35 for the isolation of gonadal PGCs (gPGCs) and embryonic skin tissue for embryonic fibroblast derivation. These embryonic fibroblasts can also be used for cellular reprogramming to generate green peafowl iPSCs. Both iPSCs and ESCs can potentially serve as the sources for generating an entirely new animal body. In this case, it can be achieved through transplantation into a sterile recipient egg, where the original blastodermal cells have already been irradiated and removed. Green peafowl PGCs can also be transplanted onto recipient eggs to create chimeric males and females, which can later cross to produce offspring, some of which may potentially be purebred green peafowl. The collection of different types of genetic resources, such as iPSCs, ESCs, PGCs, and fibroblasts, can be stored in a biobank for long-term preservation.

Natural reproduction with assisted mating is insufficient to provide fertile offspring of green peafowls, risking the extinction of the pure breed. Assisted reproductive technology (ART), such as semen collection and artificial insemination, has been an approach to prevent the extinction of non-domestic avian species [68]; however, due to the quality of sperms and the limitation of a source of pure breed for green peafowls, current stem cell technology becomes a promising approach to support the ART approaches by gaining more viable cells with high potency and the capability of generating the whole animal body, called as pluripotent stem cells (PSCs). In mammals, PSCs are well established by the derivation of embryonic stem cells (ESCs) from the inner cell mass of the blastocyst stage in mouse and human, epiblast stem cells (EpiSCs) from epiblast of a post-implantation murine embryo, and embryonic germ cells (EGCs) from mouse and human primordial germ cells (PGCs) [69,70]. PSCs can also be generated via the cellular reprogramming of somatic cells, such as embryonic fibroblasts or adult fibroblasts, by the prolonged overexpression of reprogramming factors (which generally act as transcription factors, including OCT4 (POU5F1), SOX2, KLF4, and CMYC (OSKM), with or without NANOG, GLIS1, LIN28, or KLF2, or replacing CMYC with other reprogramming factors) [71,72,73,74,75,76]. These PSCs can differentiate into all body cell types, including ectoderm, mesoderm, endoderm, and germ cells. In avian species, the derivation of PSCs has already been under several investigations [17,77,78,79,80,81]. Chicken ESCs are the first PSCs that can be established, exhibiting similar features to mouse ESCs [82]. PGCs in chicken embryos are separated from somatic cells earlier than the mammals, and the circulating PGCs extracted from chicken blood can be maintained in culture for an extended period [78,83,84]. Due to the high cell potency of PSCs, obtaining PSCs from the endangered peafowls will support the conservation program tremendously by giving rise to whole new offspring based on the injection of the PSCs into better recipient avian eggs with perhaps similar size of egg and yolk content in the similar approaches used to generate the transgenic and chimeric chickens [85] (Figure 2). Additionally, enhancing embryonic viability during iPSCs injection into stage X embryos, maintaining a small window (<0.5 cm diameter) after eggshell windowing, and loading 1 × 10^4^ cells with an injection volume under 2 µL to minimize embryonic mortality in a larger volume is lethal [21]. 

How can PSCs be derived from the green peafowls? Two sources, including (1) blastodermal cells after the egg is laid as ESCs [86] and (2) iPSCs from differentiated cells (embryonic and adult sources), are the best for PSC derivation [20,21]. Based on the technique used for chicken ESCs derivation [77,86], hypothetically green peafowl ESCs can also be derived from an equivalent EGK X stage after freshly laid eggs. Alternatively, more embryonic fibroblasts can be obtained via the incubation of the peafowl eggs to gain an embryo at Day 13 (equivalent to Day 8–9 of a chicken embryo or HH stage 33–35) for cellular reprogramming to generate iPSCs. However, this seems not to be practical in the case of low fertile eggs, and only a few embryos can be developed each year (personal communication). Alternatively, adult fibroblasts from green peafowl can also be derived from feathers and skin tissues. It has already been shown that the feather cells can be used to generate iPSCs from avian species [17,87] and this could be applied also to green peafowls. Using a robust method for reprogramming from mouse and human models including retrovirus-based pMXs vector carrying mouse OSKM or Sendai virus-carrying human OSKM cannot support the long-term culture of chick reprogrammed cells while using chicken homologs of OSKM, including POU5F3, SOX2, KLF4, and CMYC with the NANOG support long-term culture of the reprogrammed cells [88]. Thus, it seems that avian transcription factors are better to induce avian pluripotency, and that NANOG is essential to facilitate pluripotency acquisition. However, a recent study using more reprogramming factors from mouse sequences including modified Oct4 (Pou5f1) with MyoD-derived transactivation domain and mouse Sox2, Klf4, C-myc, Nanog, Klf2, and Lin28 supports the induction of iPSC generation from chicken, Okinawa rail, Japanese ptarmigan, and Blakiston’s fish owl [17]. This suggests that avian pluripotency acquisition can probably be enhanced by adjusting reprogramming factor choices, and that mouse–chicken transcription factors exhibit conserved sequences sufficient to gather a cross-species network of pluripotency. Thus, the requirement of species-specific transcription factors is needed to be explored in further studies. In addition, a mammalian iPSC induction medium containing human LIF, 2i (PD0325901 and CHIR99021), basic FGF with ROCK inhibitor, thiazovivin can be used to induce various endangered avian iPSCs [17,21]. This recent advance in iPSC generation attempts in avian species provides a possible strategy to induce iPSCs from endangered green peafowl. The first thing to consider is the phylogeny of the green peafowl to the avian species that have already been studied for pluripotency induction. As mentioned earlier, green peafowl is in the order Galliformes, and thus, the strategy used in the chicken model should be able to be applied in the peafowl species in terms of both choices of reprogramming factor cocktail and induction/maintenance media. To support the characterization of iPSCs from green peafowl, obtaining ESCs from the early embryos of green peafowl to be used as a positive control should be performed; however, due to the limitation of getting fertilized eggs from the green peafowl, blue peafowl ESCs can be used as an alternative. In addition to securing the genetics of green peafowl, the generation of PSCs will be a great tool to understand the development of the peafowls from a differentiation approach toward genetic diversity and background as we will have more resources of cells to investigate, allowing for work on a transcriptomic comparison between peafowl and closely related species to analyze the conserved and diverge expression during differentiation to explore the timeframe of phenotypic distinction. 

## 6. Conclusions

The conservation of endangered avian species, particularly green peafowl, is a challenging endeavor due to several issues contributing to their population decline. Understanding the biology of green peafowls’ reproduction in comparison with closely related species such as blue peafowl is advantageous for overseeing species restoration by utilizing assisted technologies with their interspecific counterparts. Avian omics resources provide researchers with in-depth insights into the genetic diversity and distribution of wild green peafowl. They also shed light on evolutionary changes in genetic repertoire, which is crucial for understanding why green peafowls are more susceptible to endangerment in their natural habitats. These databases serve as a valuable resource for shaping conservation strategies. In addition, the revival and restoration of green peafowl species can be achieved through assisted reproductive technologies (ARTs). Another potentially more practical and promising method involves stem cell-mediated technology, specifically iPSCs. It offers an alternative tool for generating new offspring of endangered peafowl by in vivo conservation. Nevertheless, there are potential challenges that need to be addressed, such as ensuring a reliable source of the purebred fertilized eggs for embryonic manipulation and conducting further investigation into the feasible protocol for deriving iPSCs as biobanks from different sources of somatic cells. The goal is to eventually overcome these limitations so that the restoration of green peafowl species can be accomplished in the future.

## References

[1] IUCN (2020). www.iucn.org/about.

[2] Singh B., Mal G., Gautam S.K., Mukesh M. (2019). Biotechnology for Wildlife. Advances in Animal Biotechnology.

[3] Comizzoli P. (2015). Biotechnologies for wildlife fertility preservation. Anim. Front..

[4] Barna J., Végi B., Liptói K., Patakiné Várkonyi E., Presicce G.A. (2020). Chapter 13—Reproductive technologies in avian species. Reproductive Technologies in Animals.

[5] Comizzoli P., Holt W.V. (2019). Breakthroughs and new horizons in reproductive biology of rare and endangered animal species. Biol. Reprod..

[6] Bolton R.L., Mooney A., Pettit M.T., Bolton A.E., Morgan L., Drake G.J., Appeltant R., Walker S.L., Gillis J.D., Hvilsom C. (2022). Resurrecting biodiversity: Advanced assisted reproductive technologies and biobanking. Reprod. Fertil..

[7] Mastromonaco G.F., Songsasen N., Presicce G.A. (2020). Chapter 7—Reproductive technologies for the conservation of wildlife and endangered species. Reproductive Technologies in Animals.

[8] Sun Y., Li Y., Zong Y., Mehaisen G.M.K., Chen J. (2022). Poultry genetic heritage cryopreservation and reconstruction: Advancement and future challenges. J. Anim. Sci. Biotechnol..

[9] Tajima A. (2013). Conservation of Avian Genetic Resources. J. Poult. Sci..

[10] Jaiswal S.K., Gupta A., Saxena R., Prasoodanan V.P.K., Sharma A.K., Mittal P., Roy A., Shafer A.B.A., Vijay N., Sharma V.K. (2018). Genome Sequence of Peacock Reveals the Peculiar Case of a Glittering Bird. Front. Genet..

[11] Zhou T.C., Sha T., Irwin D.M., Zhang Y.P. (2015). Complete mitochondrial genome of the Indian peafowl (*Pavo cristatus*), with phylogenetic analysis in phasianidae. Mitochondrial DNA.

[12] Zhang X., Lin C., Li H., Liu S., Wang Q., Yang S., Shi M., Sahu S.K., Zhu Y., Wang J. (2022). Chromosome-Level Genome Assembly of the Green Peafowl (*Pavo muticus*). Genome Biol. Evol..

[13] Liu S., Chen H., Ouyang J., Huang M., Zhang H., Zheng S., Xi S., Tang H., Gao Y., Xiong Y. (2022). A high-quality assembly reveals genomic characteristics, phylogenetic status, and causal genes for leucism plumage of Indian peafowl. Gigascience.

[14] Hohenlohe P.A., Funk W.C., Rajora O.P. (2021). Population genomics for wildlife conservation and management. Mol. Ecol..

[15] Du H.Y., Zhang X.Y., Dinh T.D., Ma Y., Zong C., Li G.L., Dahmer T.D., Xu Y.C. (2020). Identification of hybrid green peafowl using mitochondrial and nuclear markers. Conserv. Genet. Resour..

[16] Dong F., Kuo H.C., Chen G.L., Wu F., Shan P.F., Wang J., Chen D., Lei F.M., Hung C.M., Liu Y. (2021). Population genomic, climatic and anthropogenic evidence suggest the role of human forces in endangerment of green peafowl (*Pavo muticus*). Proc. R. Soc. B Biol. Sci..

[17] Katayama M., Fukuda T., Kaneko T., Nakagawa Y., Tajima A., Naito M., Ohmaki H., Endo D., Asano M., Nagamine T. (2022). Induced pluripotent stem cells of endangered avian species. Commun. Biol..

[18] Han J.Y., Lee H.C., Park T.S. (2015). Germline-competent stem cell in avian species and its application. Asian J. Androl..

[19] Rosselló R.A., Chen C.C., Dai R., Howard J.T., Hochgeschwender U., Jarvis E.D. (2013). Mammalian genes induce partially reprogrammed pluripotent stem cells in non-mammalian vertebrate and invertebrate species. eLife.

[20] Fuet A., Pain B. (2017). Chicken induced pluripotent stem cells: Establishment and characterization. Methods Mol. Biol..

[21] Lu Y., West F.D., Jordan B.J., Beckstead R.B., Jordan E.T., Stice S.L. (2015). Generation of Avian Induced Pluripotent Stem Cells. Methods Mol. Biol..

[22] Lu Y., West F.D., Jordan B.J., Mumaw J.L., Jordan E.T., Gallegos-Cardenas A., Beckstead R.B., Stice S.L. (2012). Avian-induced pluripotent stem cells derived using human reprogramming factors. Stem Cells Dev..

[23] Lu Y., West F.D., Jordan B.J., Jordan E.T., West R.C., Yu P., He Y., Barrios M.A., Zhu Z., Petitte J.N. (2014). Induced pluripotency in chicken embryonic fibroblast results in a germ cell fate. Stem Cells Dev..

[24] Yu P., Lu Y., Jordan B.J., Liu Y., Yang J.Y., Hutcheson J.M., Ethridge C.L., Mumaw J.L., Kinder H.A., Beckstead R.B. (2014). Nonviral minicircle generation of induced pluripotent stem cells compatible with production of chimeric chickens. Cell. Reprogram..

[25] Mukhtar N., Ahmad T., Zee-Waqar M. (2022). Exotic avian species. Reference Module in Food Science.

[26] Rajpoot A., Kumar V.P., Arunachalam K., Rasaily S.S. (2021). National bird, Indian peafowl (*Pavo cristatus*): Using DNA technology for species identification from degraded sample from Uttarakhand, India. Forensic Sci. Int. Anim. Environ..

[27] Beebe W. (1922). A Monograph of the Pheasants.

[28] Coles B.H., Tully T.N., Dorrestein G.M., Jones A.K., Cooper J.E. (2009). 13—Galliformes. Handbook of Avian Medicine.

[29] Deignan H.G. (1963). Museum of Natural History Checklist of the Birds of Thailand.

[30] McGowan P.J.K., Duckworth J.W., Xianji W., Van Balen B., Xiaojun Y., Mohd U., Khan K.M., Yatim S.H., Thanga L., Setiawan I. (1998). A review of the status of the Green Peafowl *Pavo muticus* and recommendations for future action. Bird Conserv. Int..

[31] Sukumal N., Dowell S.D., Savini T. (2017). Micro-habitat selection and population recovery of the Endangered Green Peafowl *Pavo muticus* in western Thailand: Implications for conservation guidance. Bird Conserv. Int..

[32] Urban M.C. (2015). Climate change. Accelerating extinction risk from climate change. Science.

[33] Jose V.S., Nameer P.O. (2020). The expanding distribution of the Indian Peafowl (*Pavo cristatus*) as an indicator of changing climate in Kerala, southern India: A modelling study using MaxEnt. Ecol. Indic..

[34] Wang B., Ye W., Xu Y., Zhong X., Zhang J., Yang N., Yang B., Zhou C. (2023). Climate change affects Galliformes taxonomic, phylogenetic and functional diversity indexes, shifting conservation priority areas in China. Divers. Distrib..

[35] Li B., Liang C., Song P., Liu D., Qin W., Jiang F., Gu H., Gao H., Zhang T. (2023). Threatened birds face new distribution under future climate change on the Qinghai-Tibet Plateau (QTP). Ecol. Indic..

[36] Liu Z., Tian S., Lu S., Zhu Z., Peng Y., Li X., An L., Li J., Xu J., Wang Y. (2023). Climate and land-use changes threaten the effectiveness of protected areas for protecting Galliformes in Southeast Asia. Front. Ecol. Evol.

[37] Cao A., Shi X. (2022). The Effects of Climate Change on Habitat Connectivity: A Case Study of the Brown-Eared Pheasant in China. Land.

[38] Asgharzadeh M., Alesheikh A.A., Yousefi M. (2023). Disentangling the impacts of climate and land cover changes on habitat suitability of common pheasant Phasianus colchicus along elevational gradients in Iran. Environ. Sci. Pollut. Res..

[39] Pearce-Higgins J.W. (2021). Climate Change and the UK’s Birds.

[40] Root T.L., Price J.T., Hall K.R., Schneider S.H., Rosenzweig C., Pounds J.A. (2003). Fingerprints of global warming on wild animals and plants. Nature.

[41] Kong D., Wu F., Shan P., Gao J., Yan D., Luo W., Yang X. (2018). Status and distribution changes of the endangered Green Peafowl (*Pavo muticus*) in China over the past three decades (1990s–2017). Avian Res..

[42] Rose P. (2021). Evidence for Aviculture: Identifying Research Needs to Advance the Role of Ex Situ Bird Populations in Conservation Initiatives and Collection Planning. Birds.

[43] Green R.E., Braun E.L., Armstrong J., Earl D., Nguyen N., Hickey G., Vandewege M.W., St John J.A., Capella-Gutierrez S., Castoe T.A. (2014). Three crocodilian genomes reveal ancestral patterns of evolution among archosaurs. Science.

[44] Yonezawa T., Segawa T., Mori H., Campos P.F., Hongoh Y., Endo H., Akiyoshi A., Kohno N., Nishida S., Wu J. (2017). Phylogenomics and Morphology of Extinct Paleognaths Reveal the Origin and Evolution of the Ratites. Curr. Biol..

[45] Nagai H., Mak S.S., Weng W., Nakaya Y., Ladher R., Sheng G. (2011). Embryonic development of the emu, Dromaius novaehollandiae. Dev. Dyn..

[46] Eyal-Giladi H., Kochav S. (1976). From cleavage to primitive streak formation: A complementary normal table and a new look at the first stages of the development of the chick. I. General morphology. Dev. Biol..

[47] Hamburger V., Hamilton H.L. (1951). A series of normal stages in the development of the chick embryo. J. Morphol..

[48] Hedges S.B., Dudley J., Kumar S. (2006). TimeTree: A public knowledge-base of divergence times among organisms. Bioinformatics.

[49] Feng S., Stiller J., Deng Y., Armstrong J., Fang Q., Reeve A.H., Xie D., Chen G., Guo C., Faircloth B.C. (2020). Dense sampling of bird diversity increases power of comparative genomics. Nature.

[50] Wu L., Jiao X., Zhang D., Cheng Y., Song G., Qu Y., Lei F. (2021). Comparative Genomics and Evolution of Avian Specialized Traits. Curr. Genomics..

[51] Prum R.O., Berv J.S., Dornburg A., Field D.J., Townsend J.P., Lemmon E.M., Lemmon A.R. (2015). A comprehensive phylogeny of birds (Aves) using targeted next-generation DNA sequencing. Nature.

[52] Grzywacz B., Skórka P. (2021). Genome size versus geographic range size in birds. PeerJ.

[53] Organ C.L., Shedlock A.M., Meade A., Pagel M., Edwards S.V. (2007). Origin of avian genome size and structure in non-avian dinosaurs. Nature.

[54] Zhang G., Li C., Li Q., Li B., Larkin D.M., Lee C., Storz J.F., Antunes A., Greenwold M.J., Meredith R.W. (2014). Comparative genomics reveals insights into avian genome evolution and adaptation. Science.

[55] Graves J.A. (2014). Avian sex, sex chromosomes, and dosage compensation in the age of genomics. Chromosome Res..

[56] Smith C.A., Major A.T., Estermann M.A. (2021). The Curious Case of Avian Sex Determination. Trends Genet..

[57] Kratochvíl L., Gamble T., Rovatsos M. (2021). Sex chromosome evolution among amniotes: Is the origin of sex chromosomes non-random?. Philos. Trans. R. Soc. Lond. B. Biol. Sci..

[58] Deviatiiarov R., Nagai H., Ismagulov G., Stupina A., Wada K., Ide S., Toji N., Zhang H., Sukparangsi W., Intarapat S. (2023). Dosage compensation of Z sex chromosome genes in avian fibroblast cells. Genome Biol..

[59] Park T.S., Kang K.S., Han J.Y. (2013). Current genomic editing approaches in avian transgenesis. Gen. Comp. Endocrinol..

[60] Park J.S., Lee K.Y., Han J.Y. (2020). Precise Genome Editing in Poultry and Its Application to Industries. Genes.

[61] Lee J., Kim D.H., Lee K. (2020). Current Approaches and Applications in Avian Genome Editing. Int. J. Mol. Sci..

[62] Kim Y.M., Woo S.J., Han J.Y. (2023). Strategies for the Generation of Gene Modified Avian Models: Advancement in Avian Germline Transmission, Genome Editing, and Applications. Genes.

[63] Kang S.J., Choi J.W., Kim S.Y., Park K.J., Kim T.M., Lee Y.M., Kim H., Lim J.M., Han J.Y. (2008). Reproduction of wild birds via interspecies germ cell transplantation. Biol. Reprod..

[64] Lee J., Kim D.H., Karolak M.C., Shin S., Lee K. (2022). Generation of genome-edited chicken and duck lines by adenovirus-mediated in vivo genome editing. Proc. Natl. Acad. Sci. USA.

[65] Garda S., Schwarz J.M., Schuelke M., Leser U., Seelow D. (2021). Public data sources for regulatory genomic features. Med. Genet..

[66] Pan Z., Wang Y., Wang M., Wang Y., Zhu X., Gu S., Zhong C., An L., Shan M., Damas J. (2023). An atlas of regulatory elements in chicken: A resource for chicken genetics and genomics. Sci. Adv..

[67] Chakraborty A., Mondal S., Mahajan S., Sharma V.K. (2023). High-quality genome assemblies provide clues on the evolutionary advantage of blue peafowl over green peafowl. Heliyon.

[68] Gee G.F., Bertschinger H., Donoghue A.M., Blanco J., Soley J. (2004). Reproduction in nondomestic birds: Physiology, semen collection, artificial insemination and cryopreservation. Avian Poult. Biol. Rev..

[69] Thomson J.A., Itskovitz-Eldor J., Shapiro S.S., Waknitz M.A., Swiergiel J.J., Marshall V.S., Jones J.M. (1998). Embryonic stem cell lines derived from human blastocysts. Science.

[70] Martin G.R. (1981). Isolation of a pluripotent cell line from early mouse embryos cultured in medium conditioned by teratocarcinoma stem cells. Proc. Natl. Acad. Sci. USA.

[71] Takahashi K., Yamanaka S. (2006). Induction of Pluripotent Stem Cells from Mouse Embryonic and Adult Fibroblast Cultures by Defined Factors. Cell.

[72] Takahashi K., Tanabe K., Ohnuki M., Narita M., Ichisaka T., Tomoda K., Yamanaka S. (2007). Induction of Pluripotent Stem Cells from Adult Human Fibroblasts by Defined Factors. Cell.

[73] Wang L., Su Y., Huang C., Yin Y., Chu A., Knupp A., Tang Y. (2019). NANOG and LIN28 dramatically improve human cell reprogramming by modulating LIN41 and canonical WNT activities. Biol. Open.

[74] Maekawa M., Yamaguchi K., Nakamura T., Shibukawa R., Kodanaka I., Ichisaka T., Kawamura Y., Mochizuki H., Goshima N., Yamanaka S. (2011). Direct reprogramming of somatic cells is promoted by maternal transcription factor Glis1. Nature.

[75] Nakagawa M., Koyanagi M., Tanabe K., Takahashi K., Ichisaka T., Aoi T., Okita K., Mochiduki Y., Takizawa N., Yamanaka S. (2008). Generation of induced pluripotent stem cells without Myc from mouse and human fibroblasts. Nat. Biotechnol..

[76] Yu J., Vodyanik M.A., Smuga-Otto K., Antosiewicz-Bourget J., Frane J.L., Tian S., Nie J., Jonsdottir G.A., Ruotti V., Stewart R. (2007). Induced pluripotent stem cell lines derived from human somatic cells. Science.

[77] Pain B., Clark M.E., Shen M., Nakazawa H., Sakurai M., Samarut J., Etches R.J. (1996). Long-term in vitro culture and characterisation of avian embryonic stem cells with multiple morphogenetic potentialities. Development.

[78] Van De Lavoir M.C., Diamond J.H., Leighton P.A., Mather-Love C., Heyer B.S., Bradshaw R., Kerchner A., Hooi L.T., Gessaro T.M., Swanberg S.E. (2006). Germline transmission of genetically modified primordial germ cells. Nature.

[79] Whyte J., Glover J.D., Woodcock M., Brzeszczynska J., Taylor L., Sherman A., Kaiser P., McGrew M.J. (2015). FGF, Insulin, and SMAD Signaling Cooperate for Avian Primordial Germ Cell Self-Renewal. Stem Cell Rep..

[80] Choi H.W., Kim J.S., Choi S., Ju Hong Y., Byun S.J., Seo H.G., Do J.T. (2016). Mitochondrial remodeling in chicken induced pluripotent stem-like cells. Stem Cells Dev..

[81] Katayama M., Hirayama T., Tani T., Nishimori K., Onuma M., Fukuda T. (2018). Chick derived induced pluripotent stem cells by the poly-cistronic transposon with enhanced transcriptional activity. J. Cell. Physiol..

[82] Jean C., Oliveira N.M.M., Intarapat S., Fuet A., Mazoyer C., De Almeida I., Trevers K., Boast S., Aubel P., Bertocchini F. (2015). Transcriptome analysis of chicken ES, blastodermal and germ cells reveals that chick ES cells are equivalent to mouse ES cells rather than EpiSC. Stem Cell Res..

[83] Macdonald J., Glover J.D., Taylor L., Sang H.M., McGrew M.J. (2010). Characterisation and Germline Transmission of Cultured Avian Primordial Germ Cells. PLoS ONE.

[84] Choi J.W., Kim S., Kim T.M., Kim Y.M., Seo H.W., Park T.S., Jeong J.W., Song G., Han J.Y. (2010). Basic fibroblast growth factor activates MEK/ERK cell signaling pathway and stimulates the proliferation of chicken primordial germ cells. PLoS ONE.

[85] Zhang Y., Yang H., Zhang Z., Shi Q., Wang D., Zheng M., Li B., Song J. (2013). Isolation of chicken embryonic stem cell and preparation of chicken chimeric model. Mol. Biol. Rep..

[86] Aubel P., Pain B. (2013). Chicken embryonic stem cells: Establishment and characterization. Methods Mol. Biol..

[87] Kim Y.M., Park Y.H., Lim J.M., Jung H., Han J.Y. (2017). Technical note: Induction of pluripotent stem cell-like cells from chicken feather follicle cells. J. Anim. Sci..

[88] Fuet A., Montillet G., Jean C., Aubel P., Kress C., Rival-Gervier S., Pain B. (2018). NANOG Is Required for the Long-Term Establishment of Avian Somatic Reprogrammed Cells. Stem Cell Rep..

